# High-Risk Preterm Infant Born to a Mother With COVID-19: A Case Report

**DOI:** 10.3389/fresc.2022.862403

**Published:** 2022-05-04

**Authors:** Cibelle Kayenne Martins Roberto Formiga, Aline Helena Nascimento Veloso, Kathlen Terezinha Montes Soares Fernandes, Layra Alves Guimarães, Marla Moreira Avelar, Maja Medeiros

**Affiliations:** ^1^Department of Physiotherapy, State University of Goiás-UEG, Goiânia, Brazil; ^2^Department of Medicine, Hospital das Clínicas, Federal University of Goiás-UFG, Goiânia, Brazil

**Keywords:** SARS-CoV-2 pandemic, child development, neonatal intensive care, biopsychosocial model, resilience

## Abstract

The COVID-19 pandemic raises concerns about risks for pregnant women and fetuses, considering factors such as vertical transmission and neonatal alterations caused by maternal infection. Despite this, neuropsychomotor and functional complications in infants delivered by mothers with COVID-19 are still little studied. Thus, we aimed to describe the health history and development based on ICF (International Classification of Functioning, Disability and Health) components of a high-risk preterm infant born to a mother hospitalized due to COVID-19 complications. This case report was based on medical records, developmental assessments, and maternal reports. The infant was born at 30 weeks and 3 days, weighing 1,300 g, measuring 40 cm, and with Apgar scores of 2, 5, 6, and 7. COVID-19 test was negative 1 and 72 h after birth. Moreover, the infant had cardiorespiratory complications and hyperechogenicity of the periventricular white matter. The infant presented speech and language delays during follow-up, but neuromotor development occurred according to age. The health care and follow-up provided helped the development of resilience mechanisms by the infant and family to overcome adversities in the prenatal, perinatal, and neonatal periods. The assessments based on ICF components can contribute to future studies on this topic.

## Introduction

The coronavirus disease (COVID-19) that emerged in December 2019 became a global public health crisis and was declared a pandemic by the World Health Organization (1). Although different groups were affected, Juan et al. (2) observed that pregnant women were more likely to have severe or even fatal COVID-19 (3), mainly due to important physiological changes in the immune and cardiopulmonary systems (2).

During pregnancy, COVID-19 can cause complications for the mother and fetus (4). Recent studies showed that most pregnant women were asymptomatic or had mild symptoms. However, preterm labor and perinatal death also occurred with pregnant women with COVID-19, and 3% of pregnant women with COVID-19 required intensive care (5). Although it is still unclear whether vertical transmission (e.g., from mother to fetus through breast milk) of SARS-CoV-2 occurs, early neonatal infection due to transmission or environmental contamination should not be excluded (6).

Pregnant women with severe COVID-19 can present hypoxemia and cause fetal distress, resulting in prematurity, intrauterine growth restriction, abortion, stillbirth, or low birth weight (7). Prematurity and low birth weight interfere in the neuropsychomotor development of infants (8). Moreover, a study reported an increased prevalence of COVID-19 among preterm infants with <37 weeks of gestation (4), increasing the risk of problems regarding growth and development; therefore, justifying the need for monitoring these cases by a multidisciplinary team (9). Thus, this study aimed to describe the health history of a high-risk preterm infant born to a mother hospitalized due to complications of COVID-19 and development based on ICF components.

## Case Description

Medical records of the infant were assessed at the Hospital das Clínicas of Universidade Federal de Goiás (HC-UFG) from June 2020 to December 2021. The mother was admitted to the intensive care unit (ICU) for 15 days due to acute respiratory syndrome caused by SARS-CoV-2 (Severe deficiency in Respiratory Functions, others specified b4408.3). The infant (female) was born by cesarean section at the gestational age of 30 weeks and 3 days, weighing 1,300 g and measuring 40 cm. Apgar scores were 2, 5, 6, and 7 in the first, fifth, tenth, and 20th minutes. The infant required cardiac resuscitation, surfactant administration, and intubation. COVID-19 test was negative 1 and 72 h after birth.

Body functions and structures were evaluated using imaging and laboratory tests and medical records of the period in which the infant was in the ICU, nursery, and outpatient follow-up (Table 1). The infant developed several complications associated with body functions and structures during admission, such as respiratory distress syndrome, persistent pulmonary hypertension, pulmonary hemorrhage, moderate bronchopulmonary dysplasia, and post-intubation laryngitis. Oxygen therapy was performed using invasive mechanical ventilation, continuous positive airway pressure, and oxygen cannula. An echocardiogram showed patent foramen ovale and pericardial effusion of 3 mm. Transfontanelle ultrasonography indicated hyperechogenicity of the periventricular white matter. Evoked otoacoustic emissions and brainstem auditory evoked potentials were normal. Chest computed tomography indicated diffuse impairment of lung parenchyma with ground-glass opacities associated with consolidations in the posterior portions. Cultures were within reference values, and the infant received transfusion of packed red blood cells. Despite respiratory complications, the COVID-19 test was negative.

**Table 1:** Exams performed to assess body functions and structures.

**Exam**	**Chronological age**	**Results**
COVID-19 PCR test	1 and 72 h after birth	Negative
Chest computed tomography	6 days	Diffuse impairment of lung parenchyma with ground-glass opacities associated with consolidations in the posterior portions
Echocardiogram	15 days	Patent foramen ovale and 3 mm pericardial effusion
Transfontanelle ultrasonography	1 month and 2 days	Periventricular white matter hyperechogenicity that may be associated with prematurity or hypoxia
Brainstem auditory evoked potentials	4 months and 9 days	Normal
Evoked otoacoustic emissions	7 months, 3 weeks, and 1 day	Normal
Newborn screening test	6 months, 1 week, and 1 day	Normal
Ophthalmic	6 months, 1 week, and 1 day	Normal

The infant was discharged after 25 days in the ICU with drug prescription and oxygen therapy, weighing 3,055 g and measuring 47.3 cm. Then, she was followed up by a multidisciplinary team of pediatric pulmonologists, neonatologists, pediatricians, and physical therapists. Consultations with the team were performed every 3 months, and the family received guidance regarding home care for the child. Routine assessments indicated evolution and stabilization of respiratory functions, allowing neurological and motor development according to age and oxygen weaning (nasal cannula). Speech and language evaluations were not performed due to the lack of a speech therapist in the follow-up team of the hospital. Evaluations also provided information regarding the evolution of body functions and structures and activities and participation of the child.

The ICF is a health model that presents the concepts of functioning and disability through four interconnected domains (body structures and functions, activities and participation, environmental factors, and personal factors) and provides a conceptual framework that helps clinical reasoning during assessment and intervention in children and adolescents. Nevertheless, a combination of instruments may be required to evaluate patients since no single test encompasses all ICF components (10, 11). The evaluation of body structures and functions comprised muscle tone, tendon reflexes, and sensory functions with the help of colored and sound toys. Regarding activity, standardized evaluation instruments were applied. The Alberta Infant Motor Scale assessed motor development considering percentiles for motor development between 5 and 90th: <5th (abnormal motor performance), between 5 and 25th (suspected motor performance), and > 25th (normal motor performance). The Denver Developmental Screening Test (Denver II) screened general development and classified the child as typical development or risk of delay, whereas the Portage Guide to Early Education assessed general development according to age by verifying the percentage of performance in the domains of socialization, self-care, language, motor, and cognition. The Survey of Wellbeing of Young Children was applied to assesses wellbeing of children according to the following abilities and domains: cognitive, language, and motor abilities (Developmental Milestones questionnaire); behavioral and emotional symptoms (Baby Symptoms Checklist and Pediatric Symptoms List); behavior, learning, or development (Parents' Concerns); and stress in the family environment, including parental depression, family conflicts, substance abuse, and food insecurity (Family Questions). Results were analyzed based on scores and cut-off points of the instrument.

An experienced examiner (CKMRF) assessed the ICF activity domain using methodological criteria of each instrument. This assessment was conducted in a private room at the hospital, and interpretation of activity and participation tests followed the ICF terminology based on clinical judgment (10): no difficulty (0–4%), mild difficulty (5–24%), moderate difficulty (25–49%), severe difficulty (50–95%), and complete difficulty (96–100%).

## Results

Results are presented according to findings of evaluations, considering the biopsychosocial model of the International Classification of Functioning, Disability and Health, which includes aspects related to body functions and structure, activities and participation, and personal and environmental factors (11, 12).

In Brazil, the peak of COVID-19 pandemic occurred between April and November 2020. Despite vaccination and social isolation, Brazilians are still facing the pandemic. Even in this context, the infant received full health care free of charge by the Brazilian public healthcare service (i.e., Unified Health System).

**Figure 1:**
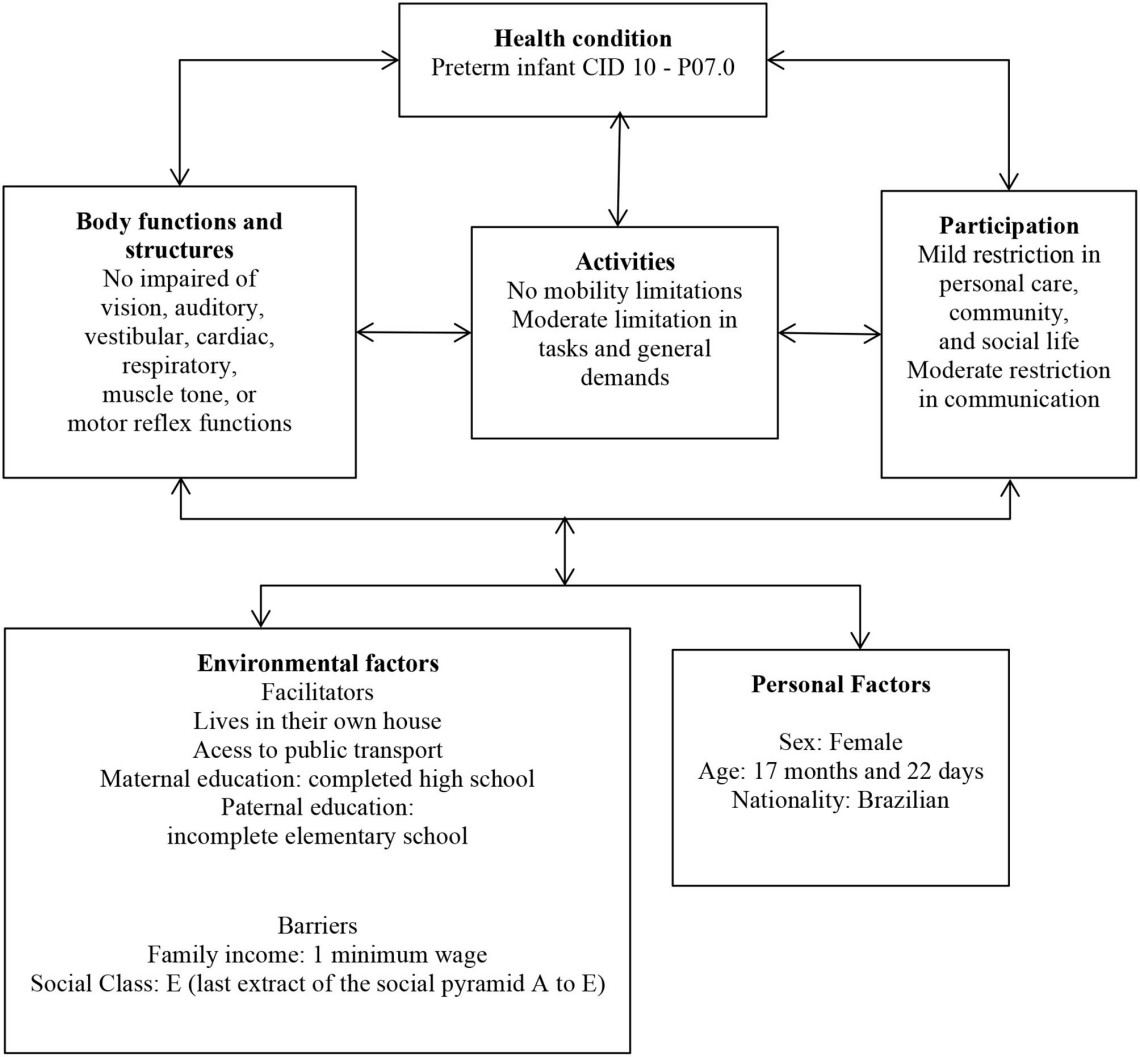
Flowchart of interactions between variables according to the International Classification of Functioning, Disability, and Health at 17 months and 22 days of age.

When this manuscript was written, the infant was 17 months and 22 days of chronological age and 15 months and 10 days of corrected age. Body functions and structures were assessed using physical and neurological exams. Activities and participation were evaluated by the Alberta Infant Motor Scale (infant motor skills in the 90th percentile), Denver Developmental Screening Test—Denver II (the infant had difficulty in items related to language, but not in personal-social, fine, and gross motor control items), Portage Guide to Early Education (94.4% in motor development, 80% in socialization, 41.6% self-help, 30% in cognition, and 16.6% in language and communication), and the Survey of Wellbeing of Young Children (minimum expected score for developmental milestones and some family complaints about social vulnerability). Personal and environmental factors (e.g., family income, parental education, and social factors that could indicate social vulnerability) were evaluated during an interview with the mother. The minimum wage in Brazil is equivalent to 221 dollars/month Findings of evaluations at 17 months and 22 days of age are shown in Figure 1.

The family received guidance regarding nutritional aspects, vaccination schedule, administration of medications, and stimulation of cognitive, language, and motor developments during all consultations at the outpatient clinic.

## Discussion

We observed that the child at 17 months and 22 days of age presented deficits in aspects related to personal care, community, and social life of the activity and participation domain of the ICF, and moderate difficulty in the area of communication. We also believe that the mobility domain was not negatively impacted by health conditions of the neonatal period. The COVID-19 pandemic raised concerns about contamination and possible sequelae. According to Cheng et al. (13), social isolation affected medical follow-up, diagnostic, and preventative exams. In Brazil, the pandemic significantly affected the monitoring of pregnant women and children. Therefore, care and prevention of motor and cognitive developmental delays were hampered and negatively impacted participation and social interaction of children (14).

Although children are not a risk group for COVID-19, pregnant and puerperal women are more susceptible to complications (15) A study evaluating 101 infants delivered by mothers with COVID-19 showed no evidence of vertical transmission (16) since only two infants presented indeterminate test results indicating low viral load. Additionally, authors observed that infants delivered by mothers with severe COVID-19 were born 1 week earlier and had a higher need for phototherapy than those delivered by mothers with mild or asymptomatic COVID-19 (mean gestational age of 37.9 vs. 39.1 weeks). These results corroborate our findings, considering that the infant tested negative for COVID-19 after birth.

A scoping review showed that only one infant out of 65 delivered by mothers with COVID-19 died from neonatal complications, possibly because of extremely low birth weight. Moreover, almost all infants tested negative for COVID-19 and did not have recurrent complications (17).

Although literature lacks sufficient evidence regarding causal relationships between COVID-19 and impaired body functions and structures and activities and participation of the infant, we believe that the pandemic is a risk factor that increases vulnerability of the infant and leads to preterm birth and complications. Moreover, families in situations of social vulnerability present personal and environmental factors that may negatively impact the development of children (14, 18). On the other hand, a follow-up may help and support the multidisciplinary team in stimulating the development of mechanisms of resilience by the infant and her family to overcome adversities in the prenatal, perinatal, and neonatal periods (19). Thus, monitoring programs for these infants may be a protective factor that allows adequate development and growth and enhances their activity and participation throughout childhood and adolescence.

Being a case report, this study has limitations in generalizing the findings to the population of newborns of COVID-19 positive mothers. We also relied solely on clinical judgment on using qualifiers as the available tools had not yet been linked to the ICF. On the other hand, this report is a demonstration how capturing information using the ICF framework results in a holistic picture of potential needs for services and supports for this vulnerable population.

## Conclusion

The infant showed adaptive development for the age and overcame impairments regarding body functions and structures. Although the infant did not present limitations in mobility, we observed mild difficulties in personal care and community life and moderate restrictions in communication, general age-appropriate tasks, and demands. The family was also referred to the speech therapist.

## Data Availability Statement

The raw data supporting the conclusions of this article will be made available by the authors, without undue reservation.

## Ethics Statement

The studies involving human participants were reviewed and approved by Universidade Estadual de Goiás (no. 42042820.8.300.5078). Written informed consent to participate in this study was provided by the participants' legal guardian/next of kin.

## Author Contributions

CF, AV, KF, and LG contributed to writing the article. CF, KF, MA, and MM contributed to assessing the infant. CF and LG contributed to interviewing family. CF, AV, KF, LG, MA, and MM contributed to reviewing and approving the final text. All authors contributed to the article and approved the submitted version.

## Funding

The research was supported by the State University of Goiás (Pro-Projects Notice 05/2021) and the Federal University of Goiás.

## Conflict of Interest

The authors declare that the research was conducted in the absence of any commercial or financial relationships that could be construed as a potential conflict of interest.

## Publisher's Note

All claims expressed in this article are solely those of the authors and do not necessarily represent those of their affiliated organizations, or those of the publisher, the editors and the reviewers. Any product that may be evaluated in this article, or claim that may be made by its manufacturer, is not guaranteed or endorsed by the publisher.
